# Design and Fabrication of a Low-Cost, Multiband and High Gain Square Tooth-Enabled Metamaterial Superstrate Microstrip Patch Antenna

**DOI:** 10.3390/mi14010163

**Published:** 2023-01-08

**Authors:** Khaled Aliqab, Sunil Lavadiya, Meshari Alsharari, Ammar Armghan, Malek G. Daher, Shobhit K. Patel

**Affiliations:** 1 Department of Electrical Engineering, College of Engineering, Jouf University, Sakaka 72388, Saudi Arabia; 2 Department of Information and Communication Technology, Marwadi University, Rajkot 360003, India; 3 Physics Department, Islamic University of Gaza, Gaza P.O. Box 108, Palestine; 4 Department of Computer Engineering, Marwadi University, Rajkot 360003, India

**Keywords:** microstrip patch antenna, metamaterial, advanced, multiband, directivity, gain

## Abstract

The manuscript represents a novel square tooth-enabled superstrate metamaterial loaded microstrip patch antenna for the multiple frequency band operation. The proposed tooth-based metamaterial antenna provides better gain and directivity. Four antenna structures are numerically investigated for the different geometry of the patch and tooth. These proposed structures are simulated, fabricated, measured, and compared for the frequency range of 3 GHz to 9 GHz. The electrical equivalent model of the split-ring resonator is also analyzed in the manuscript. The comparative analysis of all of the proposed structures has been carried out, in terms of several bands, reflectance response, VSWR, gain and bandwidth. The results are compared with previously published works. The effects are simulated using a high-frequency structure simulator tool with the finite element method. The measured and fabricated results are compared for verification purposes. The proposed structure provides seven bands of operation and 8.57 dB of gain. It is observed that the proposed design offers the multiple frequency band operation with a good gain. The proposed tooth-based metamaterial antenna suits applications, such as the surveillance radar, satellite communication, weather monitoring and many other wireless devices.

## 1. Introduction

An antenna is a prime component in all kinds of wireless communication. We are converting most wired technology to wireless, to ease equipment usage and to cover long-range communications, due to the heavy demand for multiple applications, such as mobile communication, wireless local area networks, global positioning system (GPSs), radio frequency identification (RFID) and many more. RF engineers always face challenges, such as multi-bands, wide bandwidths, high gains, and power-efficient antennas [1]. There is a need to target multiple applications with the same device. To achieve the same multiband and broadband antenna, it is helpful to cover the broader spectrum. An antenna with a low profile, a small size and broadband should be located in the front of the system to target multiple wireless communication applications [2]. One of the significant challenging parts is to design a smaller antenna, as a microstrip patch antenna is more suitable. A microstrip patch antenna is also called a printed antenna or patch antenna [3]. The advantages of patch antennas are their low cost, better reconfigurability and ease of fabrication [4]. However, due to certain drawbacks of microstrip patch antennas, such as their low gain, low bandwidth and low directivity, some improvement is required [5,6]. Multiband antennas have tremendous applications in mobile communication [7,8].

There is always controversy in bandwidths and antenna sizes. The rise of one parameter will degrade another parameter. There are many ways available to achieve a multiband operation, such as engraving slots on a patch [9], loading shorted slots with outers [10], etching unit-cells of metamaterial on/around the patch and loading it on a substrate, ground layer [11]. The explosion in cutting-edge wireless communication methods has led to a steady rise in the demand for compact mobile devices. Therefore, new approaches to the design of wireless components are needed to meet many performance requirements at once. Every wireless mobile component’s design and performance improvement currently faces significant hurdles, including the need for a compact size, lightweight, low profile and cheap cost. The antenna is one of the wireless components that will need to be improved, to keep up with the demands of modern communication networks.

Many methods are available for enhancing the bandwidth and the gain of an antenna. It can be accomplished by raising the thickness of the substrate [12], but the surface radiation is affected by changing substrate’s thickness [13]. The solution is achieved by using a substrate with a low profile [14], a different impedance matching network and a slot antenna geometry [8,15]. A novel meandered high-impedance patch antenna provides the desired multiband behavior by inserting narrow slits in the patch [16]. A broadband folded patch antenna is used for wireless local area network applications [17]. Multiple patches and ground plane slots are used to achieve the multiband operation [18]. Different shapes in the patch also enrich the antenna’s bandwidth [19,20]. There are some approaches available for the antenna’s gain improvement, such as the partial ground method, the diffractive ground [21], an artificially soft surface [22], micromachining technology and a substrate with a high dielectric constant, such as liquid (water-sea), rather than copper. However, there is a high fabrication cost for anElectronic Band Gap ( EBG), including the artificially soft surfaces and micromachining technology. The substrate multilayer provides a better bandwidth but degrades the antenna’s gain, as well as the efficiency of antenna’s structure [23]. The high gain antenna using the hexagon-shaped metamaterial elements, is presented in the article [6]. The shape variation of the metamaterial elements affects the band response [24,25]. The metamaterial concept-inspired wearable fractal antenna helps to target different IoT applications [26]. The fractal Sierpinski-shaped antenna concept helps to target multiple wireless applications with miniaturization features [27].

A superstrate provides an effective way to reduce the mutual coupling between radiating elements. It reduces the turns’ mutual coupling and supports the antenna’s directivity improvement [28,29]. There are many methods available for the antenna’s improvement, such as an array of the antenna, a surface-mounted horn antenna, composite conductors and a lens antenna [30,31,32]. At the same time, the main limitations of these designs are the large space requirements and the hardware needed for the high-gain antennas [6,33]. The listed problems are solved by adding artificial properties to the material, these are called metamaterials.

Metamaterial provides a negative value of permittivity (Ɛ) and permeability (µ) [34]. The primary elements for making metamaterial are the complementary split-ring resonators (CSRRs) and metal wires. The gap between the two terminals of a metal wire behaves as the capacitance, and their curvature shape provides an inductance effect. The size of such a structure is less than the ordinary resonating structure [35]. The metamaterial approach helps to achieve targets, due to their negative permittivity and negative permeability concept [36]. Metamaterial-based designs are used for making perfect lenses, invisibility cloaks, electromagnetic bandgaps (EBGs) and photonic bandgaps (PBGs) [37]. In antenna development, the composite electromagnetic bandgap structure (EBG) and the metamaterial inhibit the electromagnetic wave for a specific range of frequency [38,39]. This structure innovates a new way of developing compact and better performing RF components [40]. The two-dimensional EBG design confines the surface wave in a patch structure and metamaterial, enhancing the antenna’s gain, directivity and reflectance response [41]. There are a few possible ways for the miniaturization of metamaterial antennas. First is a substrate with a high permittivity, a low tangent loss and a rising thickness. Second is a zero-impedance plane, similar to the perfect electric conductor. Third is inserting an infinite impedance identical to an excellent magnetic conductor. Efficiency, in the third case, is reduced due to the dissipative loss [42,43]. Fourth is using metamaterial above a patch antenna to condense the surface waves. An array of metamaterial rings will enrich the antenna’s gain, but it also increases the size of the antenna. The solution is provided by inserting a tooth on the exterior surface of the ring [44].

The manuscript presented here represents a novel metamaterial-based multi-layered superstrate structure, to achieve the multiband operation for S, C and X frequency bands. Gain improvement has been achieved by adding a tooth to the metamaterial structure [45]. Four designs are simulated to achieve a high gain, multiband and broadband characteristics. The first design is a patch with connectors and metamaterial rings with the tooth. The second is a patch with connectors and metamaterial without a tooth. The third is a patch without connectors and metamaterial with the tooth. Fourth is a patch without connectors and metamaterial without a tooth. The charge distribution in the patch region is affected, due to the connector section of the exterior and interior patch elements. Therefore, it leads to a change in the reflectance response plot. The effect of the tooth over the metamaterial loaded structure helps to attain a better performance. All designs are compared using different substrate materials, such as Rogers RT Duroid 5880 and FR4 materials. The simulated results are compared, in terms of the number of bands, the bandwidth, VSWR, the reflectance response (*S*_11_) and gain.

## 2. Materials and Methods

**Figure 1:**
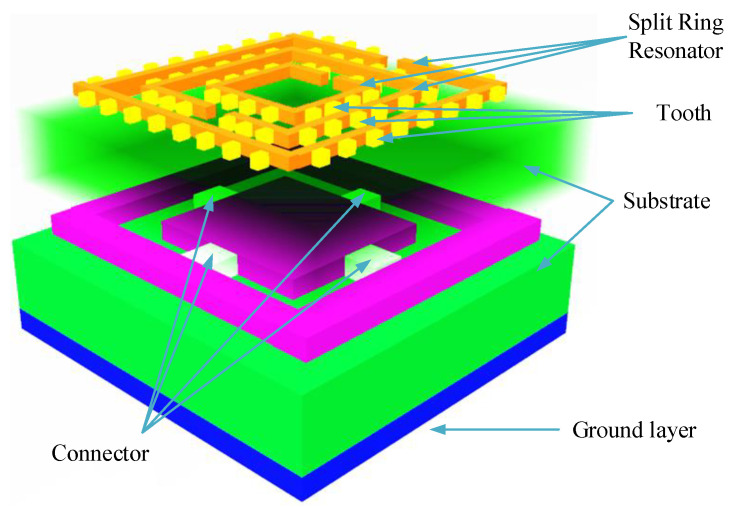
Three-dimensional interpretation of the proposed rectangular microstrip patch antenna. Two superstrate layers of substrates are used. The patch consists of a rectangular cut/cropped slot. Three SRRs with the tooth are located at the top of the upper substrate.

A 3D view of the proposed superstrate rectangular microstrip patch antenna is shown in Figure 1. The patch antenna has one rectangular cut/cropped section, which helps to achieve a multiband response and a lower reflectance response (*S***_11_**). Two substrate layers of dielectric materials are used to achieve a broader bandwidth. A novel thing introduced in the manuscript is a tooth around the split ring resonator. The design parameters of the rectangular patch antenna, include the length (L) and width (w) of the ground plane and the patch are calculated, based upon selecting the height of the substrate (h), the dielectric constant of the substrate ($\varepsilon_{r})$ and the resonating frequency ($f_{r}$) [46].

**Figure 2:**
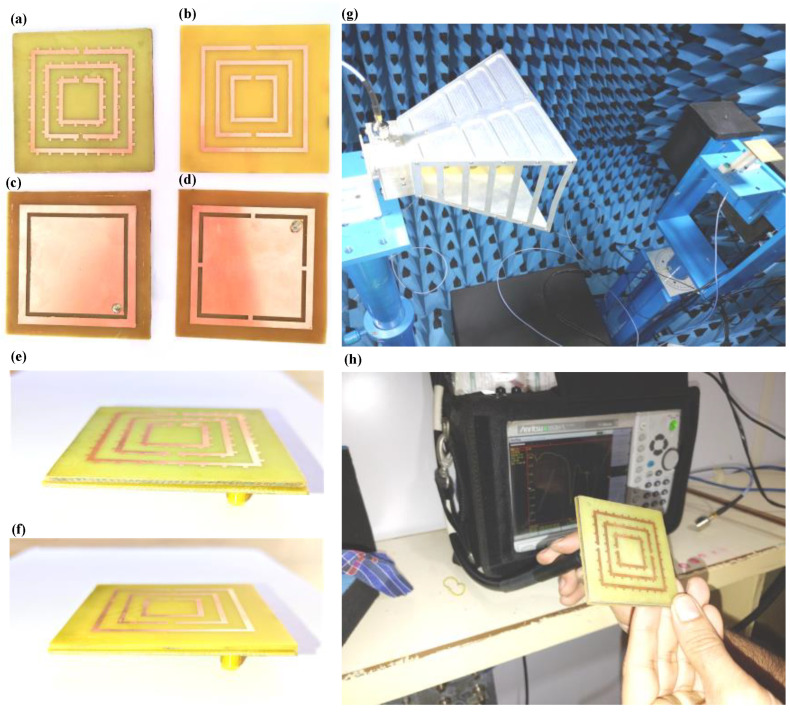
(**a**–**f**) The fabricated prototype of the proposed antenna structure. (**e**) Directivity measurement using the anechoic chamber. (**f**) Reflectance response measurement using a vector network analyzer. (**g**) Testing of antenna in Anachoic chamber. (**h**) Measuring reflectance response using Vector Network Analyser.

Figure 2 represents the fabricated prototype of the proposed antenna structure. Figure 2a represents a split ring resonator with the tooth. Figure 2b shows a split ring resonator without a tooth. Figure 2c displays the disconnected inner and exterior sides of the patch. Figure 2d represents the connected inner and exterior parts of the patch. Figure 2e,f show the side views of the proposed structure. Figure 2g shows the anechoic chamber during the antenna directivity measurement. Figure 2f displays the reflectance response measurement using a vector network analyzer.

**Figure 3:**
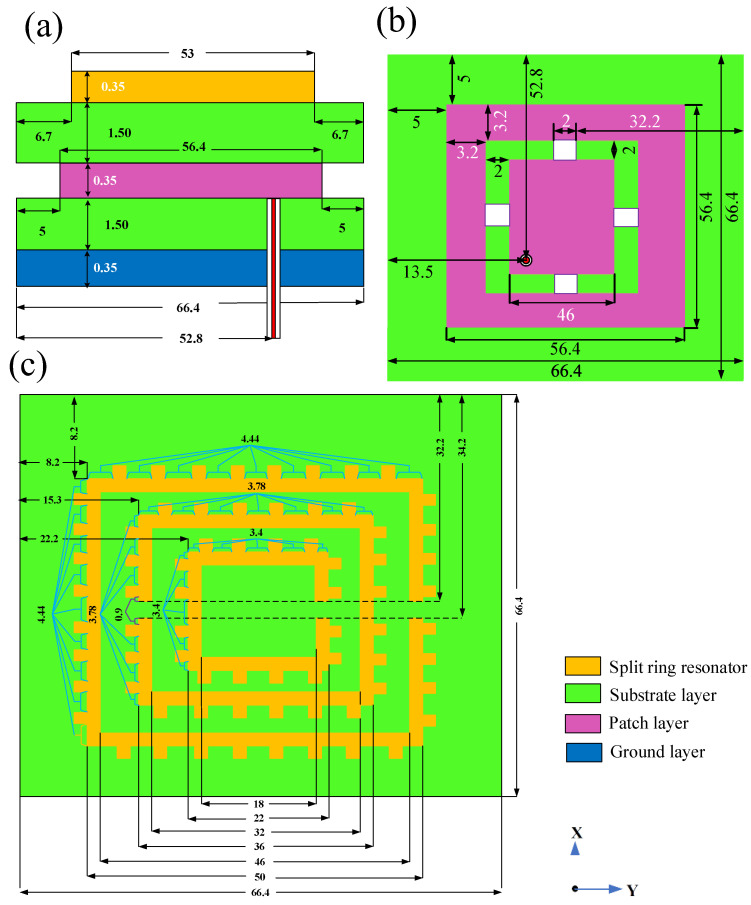
(**a**) Side view of a presented structure. (**b**) Top view of the patch. (**c**) Top view of the rectangular shaped MPA loaded with the tooth and SRR. Dimensions represented in the figure are in mm.

The side view of the designs are represented in Figure 3a. The ground layer, the patch, the split ring resonator, and the tooth thickness are 0.35 mm. The height of the substrate layer is 1.5 mm. The dimensions of the rectangular ground layer and the substrates are 66.4 mm. The patch and all of the split-ring resonators are designed in a centered position. The upper view of the antenna is represented in Figure 3b. The first (exterior), second (middle), and third (interior) split-ring resonators are positioned at 8.2 mm, 15.3 mm, and 22.2 mm, from the exterior border, respectively. The width of the SRR is 2 mm. The size of the tooth is 1.5 × 1.5 mm^2^. The gap between the two terminals of the SRR is 2 mm, and it is placed 32.2 mm away from the exterior border. The distance between the tooth located in the split-ring resonators is, respectively, 4.44 for the first (exterior), 3.78 mm for the second (middle) and 3.4 mm for the third (interior). The top view of the patch is shown in Figure 3c. The patch is kept 5 mm away from the exterior edge. The rectangular patch outer structure (POS) dimension is 56.4 mm. The cropped/cut section in the patch is 2mm. The patch interior structure (PIS) is 46 mm. The connectors for shorting the PIS with the POS are 2 mm. The connecters are kept 32.2 mm from the antenna’s exterior. The antenna’s structure is excited by applying the input from a coaxial feed. The coaxial feed is kept 52.8 mm by 13.5 mm away from the exterior. The radius of the coaxial interior is 0.12 mm, and the exterior is 0.43 mm. The ground layer, the patch, the SRR, the tooth and coaxial’s interior are made of copper. The coaxial’s exterior is made of plastic. Two types of substrate materials are considered Rogers RT Duroid 5880 (Ɛ—2.2) and FR4 (Ɛ—4.4) [47].

**Figure 4:**
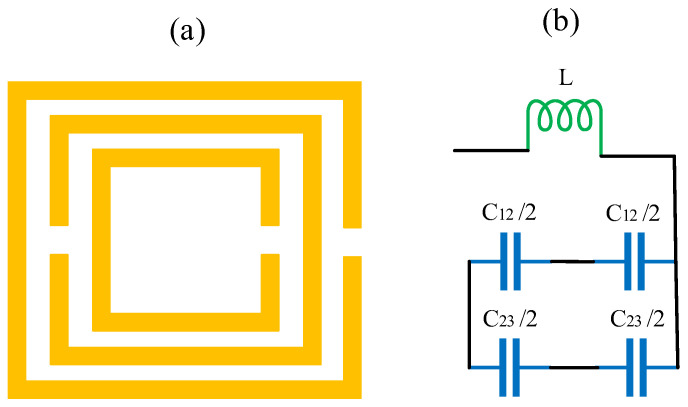
(**a**) The complementary SRR is represented. (**b**) Equivalent model of the structure.

In a SRR, the inductance effect is induced by the circular shape and the capacitance by the space is between two ring terminals. The inductance (Ls) and capacitance ($C_{s}$) per unit length can be calculated by Equations (1) and (2) [48].

(1) $$Ls=\frac{\mu_{0}b}{\sqrt{\pi }}\left[\mathrm{Log}\left(\frac{32b}{w\sqrt{\pi }}\right)-2\right]$$

(2) $$C_{s}=\varepsilon \frac{wt}{2g}$$

where, $\mu_{0}$ is the free space permittivity ($\mu_{0}=4\;\pi \times 10^{-7}$ N/$A^{2}$), the width of the ring is w, *g* is a gap between two split rings, b is the ring length, the series capacitance is $C_{s}$, t is the ring thickness. The RLC circuit of the SRR is shown in Figure 4. The resonating frequency of the proposed design can be calculated using Equation (3) [49].

(3) $$f=\frac{1}{2\pi \sqrt{L_{s}C_{s}}}$$

The performance index of the high frequency operated antenna structure can be calculate using the *S* parameters. The impedance and refractive index is calculated using the reflectance (*S*_11_) and the transmittance (*S*_21_) response using in Equations (4) and (5).

(4) $$n=\frac{1}{kd}\cos^{-1}\left[\frac{1}{2S_{21}}X\left(1-S_{11}^{2}+S_{21}^{2}\right)\right]$$

(5) $$z=\sqrt{\frac{\left(1+S_{11}^{2}\right)-(S_{21}^{2})}{\left(1-S_{11}^{2}\right)-(S_{21}^{2})}}$$

where, d is the substrate width, n is the refractive index, *k* is the wave vector and *z* is the wave impedance.

## 3. Result and Discussion

The proposed antenna design works in the range of the *S*, *C*, and *X* frequency bands. Therefore, the response analysis should be carried out by the scattering parameters (S-Parameters). The S-parameters represent the electrical behavior of a linear electrical system, describing the input and output relations among the ports of an electrical system. Unambiguously, for the high frequency, it is essential to represent a given network, in terms of waves, not by the voltage (v) or current (i).

**Figure 5:**
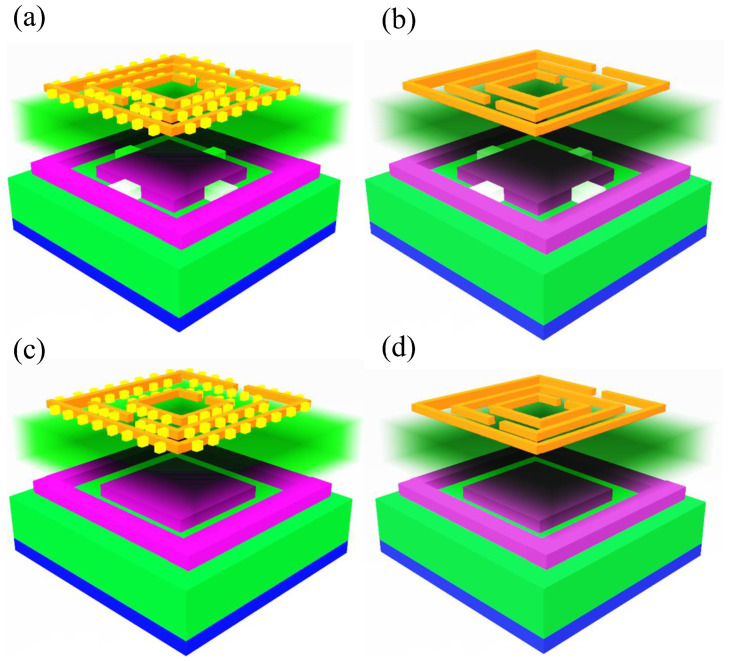
(**a**) The first design is the connected patch inner and patch outer (CPIO) and the SRR with the tooth (SWT) (**b**) Second design is the combined patch inner and patch outer (CPIO) and the SRR without a tooth (SWOT) (**c**) Third design is the disconnected patch inner and patch outer (DCPIO) and the SRR with the tooth (SWT) (**d**) Fourth design is the switch disconnected patch inner and patch outer (DCPIO) and the SRR without a tooth (SWOT).

**Figure 6:**
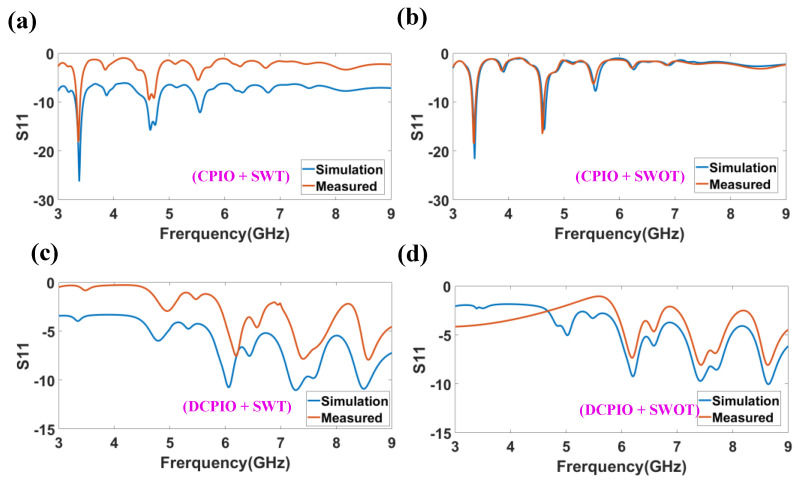
Measured and simulated reflectance response analysis using the FR-4 as substrate. The number of bands for the proposed four structures is 3, 2, 3 and 1. Minimum reflectance responses are, respectively, −26.11 dB, −21.52 dB, −11 dB and −10.10 dB. The resonance frequency is, respectively, 3.38 GHz, 3.39 GHz, 7.27 GHz and 8.64 GHz. (**a**) CPIO and SWT Design Structure. (**b**) CPIO and SWOT Design Structure. (**c**) DCPIO and SWT Structure. (**d**) DCPIO and SWOT Structure.

Figure 5 represents four antenna structures that are simulated, based upon the on and off in the interior-exterior of the patch and the tooth in the split ring resonators. The following notations were given for the design of the antenna: the connected patch interior and patch outer(CPIO), the disconnected patch interior and patch exterior (DCPIO), the SRR with a tooth (SWT), and the split ring resonator without a tooth (SWOT). The four combinations are represented in Table 1. The first structure is the CPIO and SWT. The second structure is the CPIO and SWOT. The third structure is the DCPIO and SWT. The fourth structure is the DCPIO and SWOT. The design of all of these structures is represented in Figure 6. The performance of the four designs is observed by changing both the substrate materials of Rogers RT Duroid 5880 and FR4.

*S*_11_ or gamma represents the amount of waves radiated by the antenna. The response is observed for the different frequency values. *S*_11_ is chosen for values less than −10 dB. The substrate material varies for all four structures, and the performance is analyzed and compared. The reflectance response is represented by *S*_11_. The resonating behavior of the antenna’s structure at a particular frequency is described by *S*_11_. How closely an antenna’s impedance matches that of the radio or transmission line into the load may be quantified using the voltage standing wave ratio (VSWR) value. The reflectance response and the VSWR are both directly proposed for each other. The cross-verification among both responses gives a better clarity of the proposed design. The wideband antenna transfers information over a broad range of the frequency spectrum, whereas the narrowband signals occupy a considerably smaller fraction of the spectrum and need less transmitting power for a given application. Tactical military radios, industrial monitoring, shorter-range fixed-location wireless applications, radio-frequency identification, and commercial vehicle remote keyless entry devices are all types of uses that have traditionally relied on narrowband antennas to achieve reliable links in varying operating environments. Likewise, cellular communication networks use several very narrow bands to provide a wide range of service applications. The effect of noise is also limited, due to the usage of a narrow band, compared to the wideband response.

Figure 6 represents an analysis of *S*_11_ for the simulated and measured analysis, using FR-4 material as the substrate. The similarity is observed in the measured and simulated results. There are three frequency bands observed in the CPIO and SWT antenna structures. The value of the reflectance response achieved is −26.11 dB at a resonance frequency of 3.38 GHz, and the maximum bandwidth achieved for this is 0.22 GHz. There are two frequency bands observed in the CPIO and SWOT antenna structures. The reflectance response value reached is −21.52 dB at a resonance frequency of 3.39 GHz, and the maximum bandwidth achieved for this is 0.07 GHz. There are three frequency bands observed in the DCPIO and SWT antenna structures. The value of the reflectance response achieved is −11 dB at a resonance frequency of 7.27 GHz, and the maximum bandwidth achieved for this is 0.24 GHz. There is one frequency band observed in the DCPIO and SWOT antenna structures. The reflectance response value reached is −10.10 dB at a resonance frequency of 8.64 GHz, and the maximum bandwidth achieved for this is 0.02 GHz.

**Figure 7:**
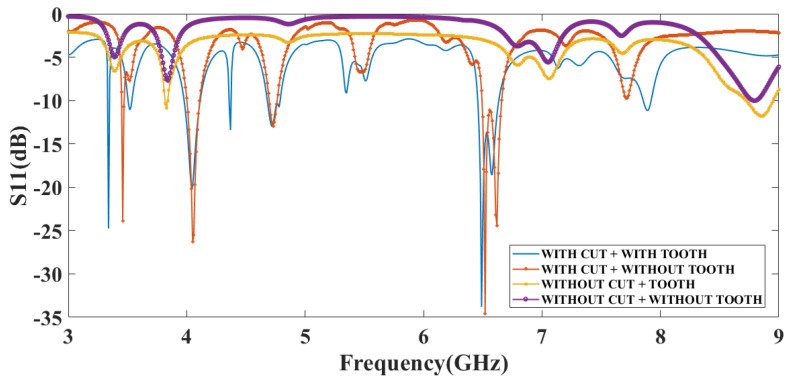
Simulated reflectance response analysis using Rogers RT Duroid as substrate. S_11_ plot for the proposed four antenna structures for the substrate Rogers RT Duroid 5880. The number of bands for the proposed four structures is 7, 6, 3 and 1. Minimum reflectance responses are, respectively, −33.79 dB, −34.54 dB, −13.79 dB and −10.05 dB. The resonance frequency is 6.49 GHz, 6.52 GHz, 8.83 GHz and 8.80 GHz, respectively.

Figure 7 represents a *S*_11_ plot by choosing the substrate as Rogers RT Duroid 5880. There are seven frequency bands observed in the CPIO and SWT antenna structures. The maximum value of the reflectance response achieved is −33.79 dB at a resonance frequency of 6.49 GHz, and the maximum bandwidth achieved for this is 0.18 GHz. There are six frequency bands observed in the CPIO and SWOT antenna structures. The maximum value of the reflectance response achieved is −34.54 dB at a resonance frequency of 6.52 GHz, and the maximum bandwidth achieved for this is 0.16 GHz. There are three frequency bands observed in the DCPIO and SWT antenna structures. The maximum value of the reflectance response achieved is −13.79 dB at a resonance frequency of 8.83 GHz, and the maximum bandwidth achieved for this is 0.51 GHz. One frequency band is observed in the DCPIO and SWOT antenna structures. The reflectance response value reached is −10.05 dB at a resonance frequency of 8.80 GHz, and the maximum bandwidth achieved for this is 0.07 GHz. Data are represented in Table 2. It is observed that more bands and minimum reflectance responses are better in CPIO and SWT antenna structures in both types of the substrate-based design. The Rogers RT Duroid-based antenna structure provides more bands and a better reflectance response than the FR4-based substrate design. Data are represented in Table 3.

**Figure 8:**
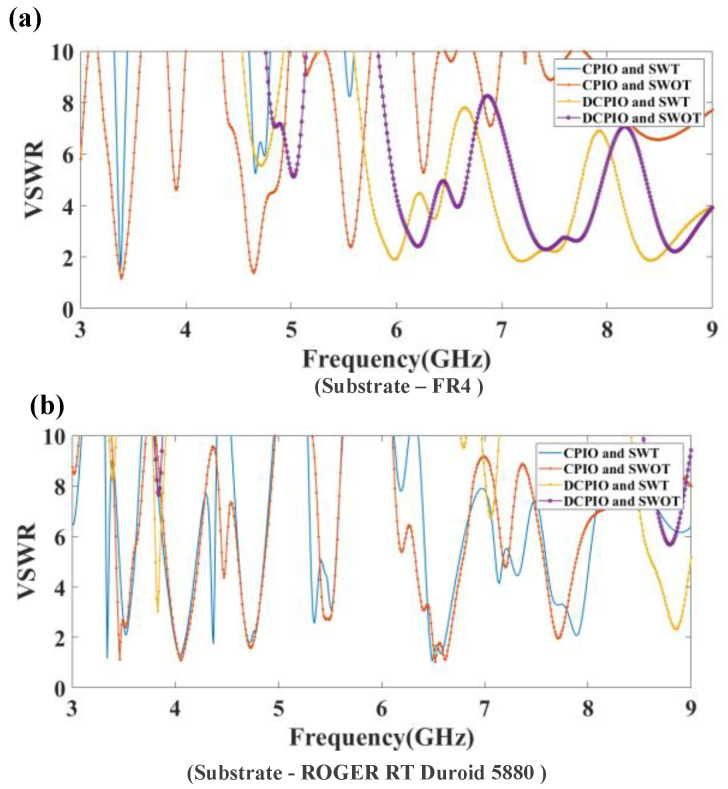
(**a**) The minimum value of the VSWR for the proposed four antenna structures by selecting the substrate as FR4, is 1.51 at 3.38 GHz, 1.18 at 3.39 GHz, 1.83 at 7.27 GHz, respectively, and 2.21 at 8.64 GHz. (**b**) The minimum value of the VSWR for the proposed four antenna structures by selecting the substrate as Rogers RT Duroid 5880 are 1.05 at 6.49 GHz, 1.03 at 6.52 GHz, 2.33 at 8.83 GHz and 5.67 at 8.80 GHz.

The voltage standing wave ratio (VSWR) represents how effectively the antenna power propagates through an antenna. Figure 8 (a) represents the VSWR response by choosing the substrate as FR4. The CPIO and SWT antenna structures provide a VSWR of 1.51 at 3.38 GHz. The CPIO and SWOT antenna structures provide a VSWR of 1.18 at 3.39 GHz. The DCPIO and SWT antenna structures provide a VSWR of 1.83 at 7.87 GHz. The DCPIO and SWOT antenna structures provide a VSWR of 2.21 at 8.64 GHz. It is observed that the connected interior and exterior patch (CPIO) design provides a better VSWR than the disconnected interior and exterior patch (DCIOP) design. Data are represented in Table 3. Figure 8b depicts the VSWR response by choosing the substrate as Rogers RT Duroid 5880. The CPIO and SWT antenna structures provide a VSWR of 1.05 at 6.49 GHz. The CPIO and SWOT antenna structures provide a VSWR of 1.03 at 6.52 GHz. The DCPIO and SWT antenna structures provide a VSWR of 2.33 at 8.83 GHz. The DCPIO and SWOT antenna structures provide a VSWR of 5.67 at 8.80 GHz. Data are represented in Table 2.

**Figure 9:**
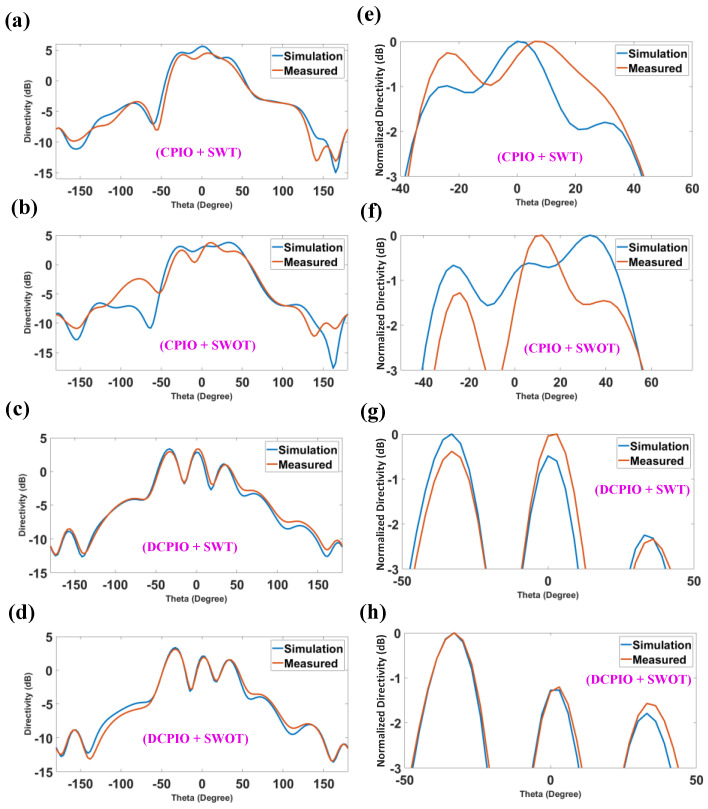
Maximum directivity and the normalized directivity for all of the modes are, respectively, 5.59 dB with 81°, 3.77 dB with 93°, 3.35 dB with 21°, 18°, 13° and 3.34 dB with 23°, 17°, 14°.

An antenna’s gain refers to how well it focuses or focuses the radio waves in a specific direction. The gain refers to an antenna’s efficiency in transforming the electrical power into radio waves in one order. In contrast, the directivity refers to an antenna’s capacity to concentrate the radiation in a single directional beam. Since many antennas and optical systems are only meant to emit electromagnetic waves in a particular direction or at a specific angle, the directivity is an essential metric to consider. Increases in directivity indicate that an antenna’s emitted signal is being focused or directed more narrowly. Increasing the beam’s directivity also increases its range. Since dBi is the standard unit of measurement for isotropic antennas, it is used to depict the antenna’s performance, compared to the isotropic antenna [50]. Figure 9 represents the 3D radiation pattern plot for the FR-4 substrate (−180° to +180°). Figure 9a,b shows the maximum directivity of 5.59 dB and the normalized directivity of 81° (−39 to +42) achieved for the CPIO and SWT modes. Figure 9c,d shows the maximum directivity of 3.77 dB and the normalized directivity 93° (−39 to +54) achieved for the CPIO and SWOT models. Figure 9e,f show that the maximum directivity is 3.35 dB and the normalized directivity is 21° (−42 to −24), 18° (−8 to +10), 13° (28 to 41) achieved for the DCPIO and SWT modes. Figure 9g,h show the maximum directivity of 3.34 dB and the normalized directivity of 23° (−46 to −23), 17° (−7 to +10), 14° (+26 to +40) achieved for the DCPIO and SWOT models.

**Figure 10:**
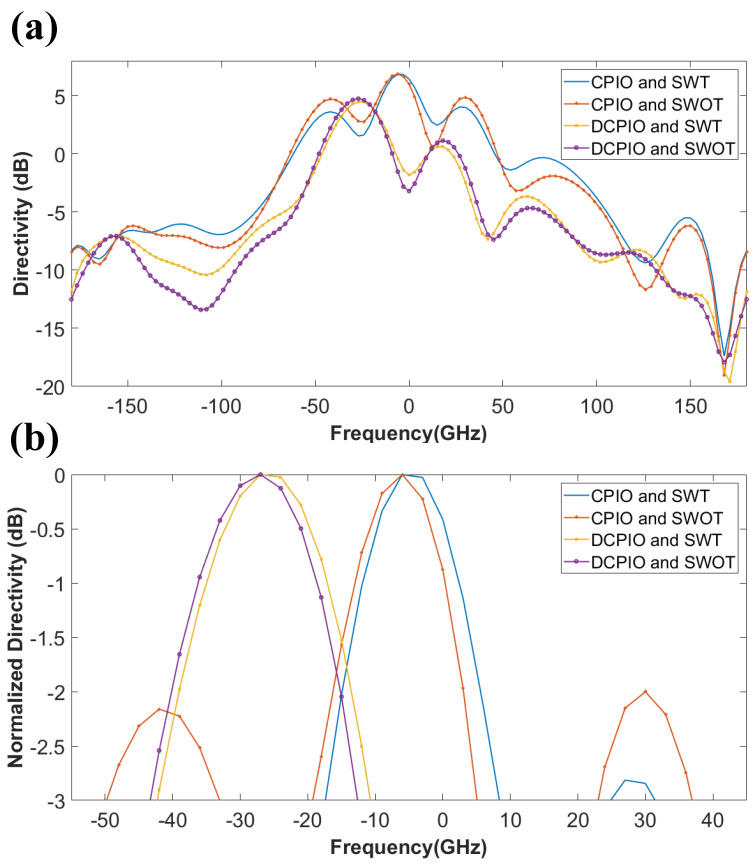
Directivity plot using the Rogers RT Duroid 5880 substrate for the range of −180° to +180°. The maximum directivity and the normalized directivity for all of the modes are 6.86 dB with 24° and 7°, 6.84 dB with 15°, 25°, 14°, and 4.47 dB with 32° and 4.74 dB with 29°.

Figure 10 represents the directivity vs. the degree plot using Rogers RT Duriod 5880 as the substrate (−180° to +180°). Figure 10a,b show that the CPIO and SWT modes represent the maximum directivity of 6.86 dB and the normalized directivity of 24° (−16 to +8). The CPIO and SWOT models represent the maximum directivity of 6.84 dB and the normalized directivity of 15° (−49 to −35), 25° (−19 to +4), and 14° (+23 to +37). The DCPIO and SWT modes represent the maximum directivity of 4.47 dB and the normalized directivity of 32° (−43 to −11). The DCPIO and SWOT models represent the maximum directivity of 4.74 dB and the normalized directivity of 29° (−43 to −14).

**Figure 11:**
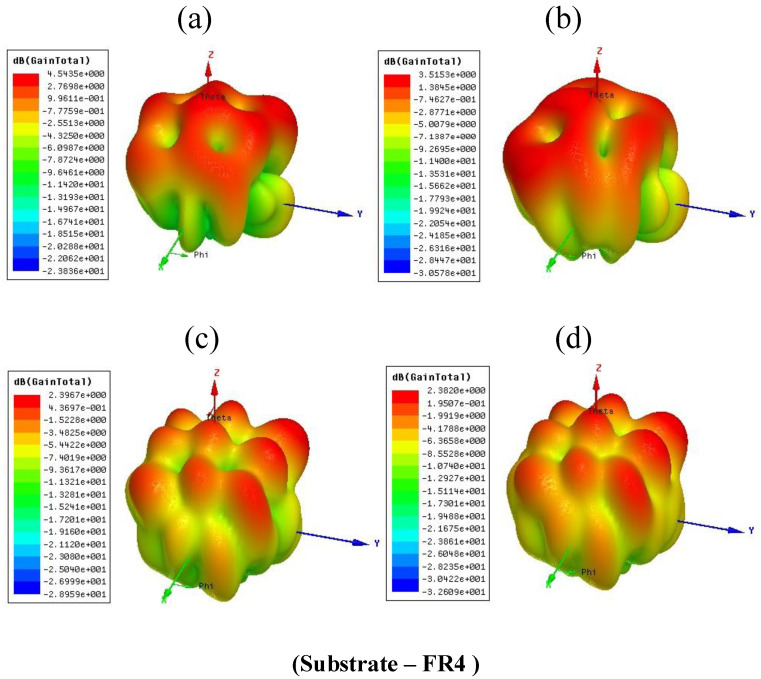
Total gain for the presented four antenna structures by selecting the substrate FR4. (**a**) 4.54 dB for the CPIO and SWT (**b**) 3.51 dB for the CPIO and SWOT (**c**) 2.39 dB for the DCPIO and SWT (**d**) 2.38 dB for the DCPIO and SWOT.

**Figure 12:**
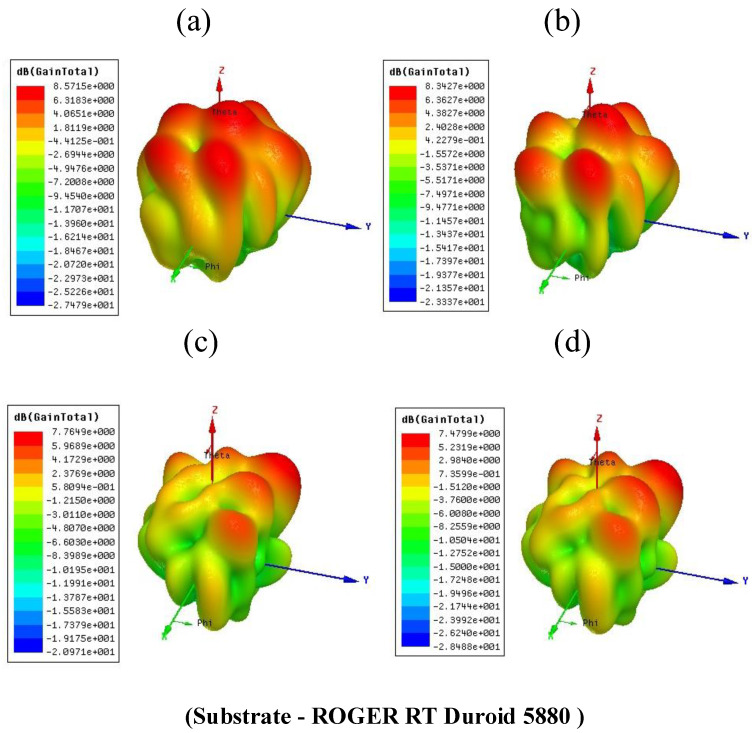
Total gain for the presented four antenna structures by selecting the substrate Rogers RT Duroid 5880. (**a**) 8.57 dB for the CPIO and SWT (**b**) 8.34 dB for the CPIO and SWOT (**c**) 7.76 dB for the DCPIO and SWT (**d**) 7.7 dB for the DCPIO and SWOT.

The antenna’s gain effectively represents the conversation of an applied electric signal into the electromagnetic waves. Figure 11 illustrates a total gain for the proposed four structures using the FR4, respectively, 4.54 dB, 3.51 dB, 2.39 dB and 2.38 dB. Figure 12 represents a total gain for the proposed four structures using Rogers RT Duroid 5880, respectively, 8.57 dB, 8.34 dB, 7.76 dB, and 7.47 dB. The comparison of the total gain for the proposed four antenna structures using the different substrate materials are represented in Table 4. The comparison of proposed design with another multiband design is represented in the Table 5. 

## 4. Conclusions

It is concluded that the tooth-added metamaterial superstrate structure with the connected interior and exterior patch provides the multiband operation with a healthy gain. The measured and fabricated results are compared for verification. The system’s performance is analyzed by varying the substrate material, inserting and removing the tooth in the split-ring resonator, and connecting and disconnecting the interior and exterior patch. The results are compared, in terms of many bands, *S***_11_**, the voltage standing wave ratio and the bandwidth for the proposed four structures, by changing the substrate material Rogers RT Duroid 5880 and FR4. The results are compared with earlier published work. The presented design represents seven frequency bands of operation, a gain of 8.57 dB, and a maximum reflectance response of −33.79 dB. The presented structure is appropriate for numerous wireless communication applications, such as radar surveillance, satellite communication and weather monitoring.

## Figures and Tables

**Table 1:** Different proposed structures.

Design	Antenna Design
1	Connected patch inner and patch outer (CPIO) and split ring resonator with tooth (SWT)
2	Connected patch inner and patch outer (CPIO) and split ring resonator without tooth (SWOT)
3	Disconnected patch inner and patch outer (DCPIO) and split ring resonator with tooth (SWT)
4	Disconnected patch inner and patch outer (DCPIO) and split ring resonator without tooth (SWOT)

**Table 2:** Reflectance plot (*S*_11_), total bands, resonance frequency, VSWR, and bandwidth data representation for the proposed four antenna structures using a substrate of Rogers RT Duroid 5880.

Design	No of Bands	Reflectance Response (*S*_11_)	Resonance Frequency (GHz)	VSWR	Bandwidth (GHz)	Starting Point (GHz)	Ending Point (GHz)
CPIO and SWT	7.00	−24.78	3.33	1.15	0.02	3.32	3.34
		−11.11	3.52	2.00	0.03	3.51	3.54
		−20.11	4.04	1.28	0.13	3.98	4.11
		−13.64	4.37	1.73	0.01	4.36	4.37
		−13.00	4.72	1.78	0.11	4.68	4.79
		−33.79	6.49	1.05	0.18	6.46	6.64
		−11.17	7.89	2.06	0.07	7.85	7.92
CPIO and SWOT	6.00	−13.37	2.91	1.54	0.03	2.90	2.93
		−23.80	3.46	1.13	0.02	3.45	3.47
		−26.30	4.05	1.10	0.11	4.00	4.11
		−12.93	4.73	1.58	0.06	4.70	4.76
		−34.54	6.52	1.03	0.16	6.50	6.66
		−10.00	7.71	1.96	0.02	7.70	7.72
DCPIO and SWT	3.00	−12.89	3.83	3.00	0.06	3.80	3.86
		−10.00	7.06	6.77	0.02	7.05	7.07
		−13.79	8.83	2.33	0.51	8.54	9.05
DCPIO and SWOT	1.00	−10.05	8.80	5.67	0.07	8.76	8.83

**Table 3:** Reflectance plot (*S*_11_), total bands, resonance frequency, VSWR, and bandwidth data representation for the proposed four antenna structures using a substrate of FR4.

Design	No of Bands	Reflectance Response (*S*_11_)	Resonance Frequency (GHz)	VSWR	Bandwidth (GHz)	Starting Point (GHz)	Ending Point (GHz)
CPIO and SWT	3.00	−26.11	3.38	1.51	0.13	3.32	3.45
		−15.70	4.66	5.21	0.22	4.59	4.81
		−12.30	5.56	8.00	0.11	5.50	5.61
CPIO and SWOT	2.00	−21.52	3.39	1.18	0.06	3.36	3.42
		−15.60	4.64	1.39	0.07	4.61	4.68
DCPIO and SWT	3.00	−10.74	6.06	1.99	0.10	6.01	6.11
		−11.00	7.27	1.83	0.24	7.17	7.41
		−10.89	8.49	1.87	0.20	8.40	8.60
DCPIO and SWOT	1.00	−10.10	8.64	2.21	0.02	8.63	8.65

**Table 4:** Total gain for the two different substrates, Rogers RT Duroid 5880 and FR4.

Design	Substrate	
	Rogers RT Duroid 5880	FR4
CPIO and SWT	8.57	4.54
CPIO and SWOT	8.34	3.51
DCPIO and SWT	7.76	2.39
DCPIO and SWOT	7.47	2.38

**Table 5:** Comparison of the proposed structure with another multiband design.

References	No of Bands	Resonating Frequency (GHz)	Minimum Reflectance Response (*S*_11_)	Peak Gain (dB)
[44]	5	4.4, 5, 5.8, 8.05	−17, −31, −13, −17	-
	4	2.9, 5.1, 5.95, 6.55, 8.3	−25, −13, −22, −25, −21.5	-
	5	3.2, 5.5, 6, 6.6, 8.3	−10, −17, −22, −12, −10	-
[9]	3	3.4, 5.7	−20, −39	-
[35]	5	5.3, 7.5, 9.8, 14.9, 19	−13, −12, −10, −23, −21	8.5
[6]	2	5.7, 10.3	−22, −21	6.38
[28]	3	4, 4.8, 9	−26, −16.5, −35	3.24
Proposed CPIO and SWT structure	7	3.33, 3.52, 4.04, 4.37, 4.72, 6.49, 7.89	−24.78, −11.11, −20.11, −13.64, −13, −33.79, −11.17	8.57

## Data Availability

The data will be made available at a reasonable request to the corresponding author.
